# Social Resources and Community Resilience in the Wake of Superstorm Sandy

**DOI:** 10.1371/journal.pone.0160824

**Published:** 2016-08-31

**Authors:** Kathleen A. Cagney, David Sterrett, Jennifer Benz, Trevor Tompson

**Affiliations:** 1 Department of Sociology, University of Chicago, Chicago, Illinois, United States of America; 2 The Associated Press-NORC Center for Public Affairs Research, NORC at the University of Chicago, Chicago, Illinois, United States of America; University of South Carolina, UNITED STATES

## Abstract

Recovery efforts after natural disasters typically focus on physical infrastructure. In general less attention is paid to the social infrastructure that might impact the capacity of the community to rebuild. We examine perceptions of preparedness and recovery (markers of resilience at the community level) in the wake of Superstorm Sandy with a novel data set that includes a multi-mode survey of twelve neighborhoods severely affected by the storm. With these data, we suggest that social resources are associated with beliefs about neighborhood resilience. People who live in communities with higher social cohesion (coefficient = .73, p <.001), informal social control (coefficient = .53, p <.001), and social exchange (coefficient = .69, p <.001) are more likely to believe their neighborhoods are well prepared for a disaster. Likewise, people living in communities with higher social cohesion (coefficient = .35, p <.01), informal social control (coefficient = .27, p <.05), and social exchange (coefficient = .42, p <.001) are more likely to be confident their neighborhoods will recover quickly from a disaster. However, the effects of social resources on beliefs about resilience vary based on neighborhood socioeconomic status (SES) and the impact of the storm. Informal social control and social exchange lead to a greater increase in confidence in recovery in low, as compared to high, SES neighborhoods. Social resources tend to have more impact on perceptions of recovery in communities less affected by the storm. In sum, these findings suggest the potential value of various forms of social intervention to better equip communities to respond when disaster strikes.

## Introduction

The relatively recent increase in climate-related disasters has drawn attention to variation in recovery, both for individuals and their communities [1,2]. On the individual level, property damage coupled with a delay in insurance remuneration could mean a significant shift in the economic well-being of the household. On the community-level, deteriorated roadways, delayed utility resumption, and divergent views about responsibility for rebuilding stymie efforts to restore neighborhoods. In general, the focus of recovery efforts typically centers on physical infrastructure and the financial resources to rebuild more broadly. Less attention is paid to the social infrastructure, such as the connectedness of community members and the social resources that emerge from it, that might impact the capacity of the community to rebuild [3].

In non-disaster circumstances, social resources at the community-level (e.g., social cohesion, informal social control, social exchange) are associated with lower crime rates, higher levels of physical activity, and a general sense of emotional well-being [4,5]. We know from earlier work that formal resources intended to provide aid in the wake of disaster are finite; a limited number of first responders can mean that community members must turn to one another to meet immediate needs and, indeed, may maintain those connections as they continue to recover [6,7]. Thus, informal sources of support might not only bear the brunt of recovery in the short-term [8], but may indeed be influential over time as a community seeks to restore itself or builds capacity for the next crisis.

Are social resources a factor in explaining why some communities recover more quickly than others, or, in other words, are resilient in the face of natural disaster? We seek to understand how communities respond to the challenges of rebuilding and how their responses vary by the social resources that characterize them. Isolating the role of social resources also allows for comparisons across communities that differ economically; this is important in the context of a natural disaster given economic resources are believed to be a key determinant of recovery and its timing. Thus, exploring social resources in the presence, and absence, of economic means is a key feature of this work. We pursue this research with a novel data source produced by the Associated Press-NORC Center for Public Affairs Research for their study “Two Years after Superstorm Sandy: Exploring Resilience in Twelve Neighborhoods.” The study included a multi-mode survey of 12 neighborhoods most affected by Superstorm Sandy. To our knowledge, no other data source captures perceptions of recovery, preparedness, and the social fabric of the community at the neighborhood level in the post-disaster context. These data provide insight into recovery, the factors that influence it, and the characteristics of communities that lead them to believe they can yet again manage a natural disaster.

## Background

Scholars have long recognized the importance of social connections to the stability and outcomes of communities [9–11]. Two characteristics of communities, collective efficacy and social exchange, research suggests, are key determinants of a community’s capacity to manage problems or implement shared goals [12–15].

Collective efficacy expands on the basic concepts of social networks and social capital and emphasizes the importance of both mutual trust and expectations of action in service to collective goals [12,13]. Collective efficacy includes two component dimensions: social cohesion and informal social control. Social cohesion refers to a sense of trust and solidarity among community members. Social cohesion is more than a network of personal connections and involves a broader sense of attachment to the community [13]. It can be seen as reflecting a normative orientation toward the community and a belief about how the community might collectively respond in time of need. Informal social control refers to residents’ willingness to intervene on behalf of each other and the ability of a community to regulate its members. Informal social control differs from formal regulations or enforcement by community institutions such as the police; it involves informal approaches that residents take to achieve public order [12]. The combination of mutual trust (social cohesion) and willingness to act (informal social control) provides the basis for a community’s collective efficacy and capacity to achieve common goals. Collective efficacy is goal-specific, and the ability of a community to accomplish one goal (e.g., lower crime) could differ from its capacity to accomplish another goal (e.g., enhance the health of residents), although such capacities are often highly correlated [16].

Social exchange, in contrast, focuses on actual interactions between residents of a community [17]. This concept measures the frequency of exchanges between residents and includes both social interactions and incidents of residents’ helping one another [14]. Social exchange assesses both community-level support and sociability in a neighborhood [18]. We distinguish between collective efficacy and social exchange because the first is about expectations for action and the other about exchange-based behavior. One could have beliefs about collective goals without interacting with neighbors in a day-to-day manner. And, one could exchange favors with a neighbor absent of the belief that the whole community would come together, either for more perfunctory tasks or in times of crisis.

Research suggests that levels of both collective efficacy and social exchange are associated with a wide variety of community outcomes including violent crime [12], and individual-level outcomes such as sexual behavior [19] and physical health [13]. In some instances these two phenomena behave differently [5], suggesting that they may perform diverse functions in response to need. In more recent work, scholars hypothesize that social resources such as community-level social capital could help neighborhoods not only in periods of relative calm but during, and in the aftermath of, natural disasters [20].

The apparent proliferation and intensity of natural disasters has led policymakers and scholars to explore the factors that lead some neighborhoods to recover more quickly and completely than other neighborhoods. A related concept to collective efficacy and social exchange, resilience, has emerged as a potential frame to understand disaster recovery at the community level. While there are a number of working definitions we note that the United Nations’ description emphases the “capacity of a system, community or society potentially exposed to hazards to adapt, by resisting or changing in order to reach and maintain an acceptable level of functioning and structure” [21]. We also note that, a decade ago, international policymakers at the 2005 World Conference on Disaster Reduction concluded that the meeting “underscored the need for, and identified ways of, building the resilience of nations and communities to disasters” [21]. Resilience may encompass multiple dimensions, including both individuals’ psychological health and a community’s economic and commercial services, infrastructure systems, and regular operation of public safety and government [22]. Norris and colleagues [6] suggest that community resilience is not a static characteristic of a community or a particular outcome, but rather it is a process of adaptation based on dynamic resources and capacities.

A select number of studies have begun to more carefully explore the relationship between social resources and resilience [23–25]. Most notably, Aldrich’s [23] work suggests that high voting rates, participation in voluntary organizations, and high levels of trust increased community resilience in neighborhoods recovering from such weather-related disasters as the 1923 Tokyo Earthquake, the 1995 Kobe Earthquake, the 2004 Indian Ocean Tsunami, and Hurricane Katrina in 2005. Several recent studies also have examined how social resources such as social capital relate to disaster preparedness [26,27]. The existing literature on resilience and disaster preparedness, however, relies mainly on broad measures of social capital such as group membership or trust [23,24,27], making it difficult to assess the social precursors to a resilient reaction. An additional set of studies relies on ethnographic or qualitative findings and, although rich in content, lacks more systematic evidence linking social resources with resilience [28–30]. As a result, there is a dearth of research that examines specific social resources at the neighborhood level and their potential contribution to resilience downstream.

**Fig 1:**
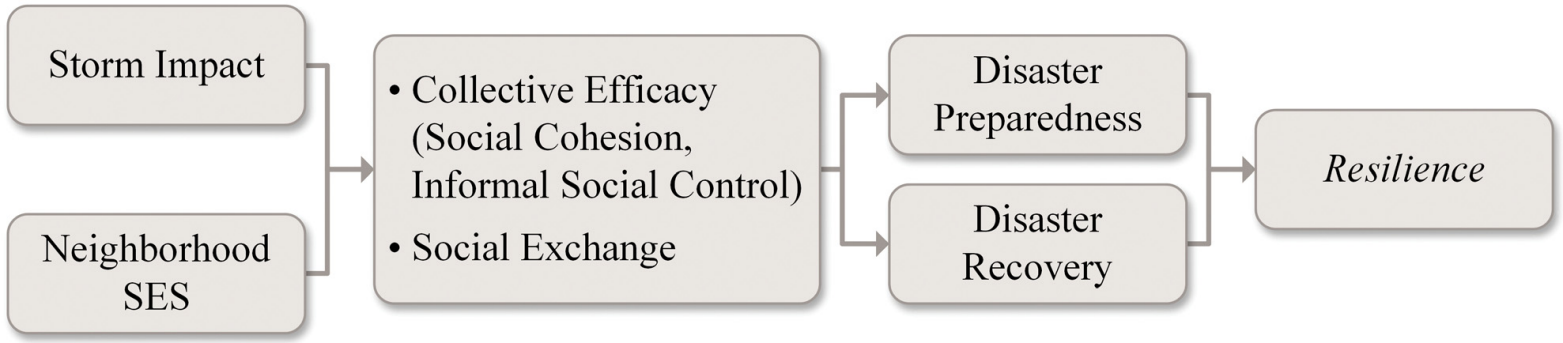
Conceptual model of social resources and their relationship to resilience.

We contribute to this literature by theorizing about two aspects of social resources, collective efficacy (i.e., social cohesion and informal social control) and social exchange, and the extent to which they contribute to resilience. In Fig 1 we provide a heuristic to describe the relationships among our key concepts of interest. In this conceptualization we see resilience as a latent construct (indicated by *italics*) with perceptions of preparedness and recovery as potential components of it. We view collective efficacy and social exchange as social resources that may be associated with these perceptions (with storm impact and neighborhood SES as important precursors). Our general aim is to examine resilience in the wake of Superstorm Sandy, unpacking the components of social resources that might contribute to it. Specifically, we examine the extent to which communities with higher levels of collective efficacy and social exchange will feel better prepared to face another disaster and better equipped to recover. We examine the two facets of collective efficacy—social cohesion and informal social control—separately, in an attempt to gain a more fine-grained insight into how each might contribute to beliefs about preparedness and recovery. We explore the extent to which these processes operate in a similar manner across low and high socioeconomic status (SES) communities. We further explore the extent to which these associations might be moderated by the storm’s impact. We hypothesize the following: 1) both collective efficacy and social exchange will be associated with perceptions of preparedness and recovery; 2) this association will be more pronounced in communities of lower SES; and 3) these social resources will be more relevant when the impact of the storm is greater. Our focus on the social component of recovery is meant to extend our understanding of what communities may be able to do to best prepare for natural disasters.

## Methods

### Data and Measures

Our data source is a multi-mode survey of 1,009 residents of twelve neighborhoods in New York and New Jersey that were affected by Superstorm Sandy [8]. The Associated Press-NORC Center for Public Affairs Research conducted the survey between June 28 and September 9, 2014. The sample included 300 respondents who completed the survey via the web, 316 who completed by telephone, and 393 respondents who completed in-person interviews. All survey modes were offered in both English and Spanish, depending on respondent preference. All telephone and in-person interviews were completed by professional interviewers employed by NORC. NORC at the University of Chicago Institutional Review Board approved this study and all data collection approaches. We adhered to the following procedures: 1) phone respondents provided informed consent after the text was read by the NORC telephone interviewer; 2) field respondents provided informed consent after the text was read by the NORC field interviewer; 3) web respondents gave consent through the web screen. Interviewers recorded verbal consent, and respondents reported consent on the web survey. The IRB approved a waiver of written consent because our research team demonstrated that the research involved no more than minimal risk of harm and involved no procedure for which written consent is normally required outside of the research context.

The 12 neighborhood areas in New York and New Jersey were selected using a two-by-two design with median household income (measured using data from the U.S. Census Bureau via the American Community Survey) and recovery status (which was measured using prior survey data, the AP-NORC Center for Public Affairs Research 2013, and key informant interviews) as central selection factors. The survey was conducted using a multi-mode address-based sample (ABS) design, and the sampling frame was based on an extract of the U.S. Postal Service delivery-sequence file. The multi-mode approach included field interviewers who targeted those who did not respond to the survey via the web or telephone. The field interviewers increased the response rate, especially among harder to reach populations such as those living in lower income or more racially diverse neighborhoods.

The final response rate was 24 percent, based on the American Association of Public Opinion Research (AAPOR) Response Rate 3 method. Sampling weights were calculated to adjust for sample design aspects (such as unequal probabilities of selection, number and type of contact attempts) and for nonresponse bias arising from differential response rates across various demographic groups. Poststratification variables included age, sex, race, and education. The weighted data, which reflect the population of the twelve neighborhoods, were used for all analyses.

#### Dependent variables

*Perceptions of disaster preparedness*. Respondents’ beliefs about their neighborhood’s preparedness for a disaster provided a measure of community resilience. Respondents were asked: “In general, how well prepared do you think your neighborhood is to handle a major disaster if it were to happen today? Would you say your neighborhood is extremely well prepared, very well prepared, moderately well prepared, not too well prepared, or not prepared at all?” [Half of respondents received the response options in reverse order]. Answers were coded 1 for not prepared at all/not too well prepared, 2 for moderately well prepared, and 3 for very well/extremely prepared.

*Perceptions of disaster recovery*. Respondents’ confidence that their neighborhoods would recover quickly from a future disaster offered another measure of community resilience. Respondents were asked: “How confident are you that your neighborhood would recover quickly after a major disaster in the future? Would you say you are extremely confident, very confident, moderately confident, not too confident, or not at all confident?” [Half of respondents received the response options in reverse order]. Answers were coded 1 for not at all confident/not too confident, 2 for moderately confident, and 3 for very/extremely confident.

We view these two dependent variables as associated with, or precursors to, the construct of resilience. The correlation between the two measures was 0.48.

#### Independent variables

*Social cohesion*. Social cohesion refers to the level of trust and solidarity in a neighborhood. A five-item factor score for social cohesion was calculated for each respondent. All respondents were asked: “Now I’m going to read some statements about people in your neighborhood. Thinking about what your neighborhood is like now, after Superstorm Sandy, please tell me whether you strongly agree, agree, disagree or strongly disagree. How about…?”

- “This is a close-knit neighborhood”
- “People around here are willing to help their neighbors”
- “People in this neighborhood generally don’t get along with each other”
- “People in this neighborhood do not share the same values”
- “People in this neighborhood can be trusted”

These items have been used in several past studies to measure social cohesion [12,13]. The factor scores ranged from -1 (low social cohesion) to 1 (high social cohesion). The alpha score for the five items was .80, and a factor analysis indicated that the five items had an Eigenvalue of 2.26.

*Informal social control*. Informal social control refers to an expectation of action within a community, and is meant to reflect the degree to which neighbors engage in informal monitoring. A five-item factor score for informal social control was calculated for each respondent. All respondents were asked: “For each of the following, please tell me if it is very likely, somewhat likely, somewhat unlikely, or very unlikely that people in your neighborhood would act in the following manner. How about…?

- “Do something about it if a group of neighborhood children were skipping school and hanging out on a street corner”
- “Do something about it if some children were spray-painting graffiti on a local building”
- “Scold a child who was showing disrespect to an adult”
- “Break up a fight in front of your house in which someone was being threatened or beaten”
- “Organize to try to do something to keep the fire station open if, because of budget cuts, the fire station closest to your home was going to be closed down by the city”

Several previous studies have used these items to measure informal social control [12,31]. The factor scores ranged from -1 (low informal social control) to 1 (high informal social control). The alpha score for the five items was .79, and a factor analysis indicated they had an Eigenvalue of 2.08. We note that both social cohesion and informal social control comprise collective efficacy. In this case we chose to examine collective efficacy’s components separately, in keeping with our aim to understand how specific perceptions of connectedness could inform preparedness and recovery.

*Social exchange*. Social exchange refers to personal interaction in a neighborhood. A five-item factor score for social exchange was calculated for each respondent. All respondents were asked: “Now I am going to ask you about some things that you might do with people in your neighborhood. For each statement, please tell me whether they happen often, sometimes, rarely, or never.”

- “Do favors for each other such as watching each other’s children or lending garden or house tools?”
- “Watch over a neighbor’s property when he or she is not home?”
- “Ask each other for advice on personal things like child rearing or job openings?”
- “Have parties or neighborhood get-togethers?”
- “Visit each other’s homes or visit on the street?”

Previous research has employed these items to measure social exchange (14). The factor scores ranged from -1 (low social exchange) to 1 (high social exchange). The alpha score for the five items is .83, and a factor analysis indicated they had an Eigenvalue of 2.4. See Table 1 for the means, standard deviations, and factor loadings for the variables described above.

**Table 1:** Descriptive statistics for key dependent and independent variables.

Variable	Mean	Standard Deviation	Factor loading
***Resilience Outcomes***			
Prepared for a disaster (N = 966)	1.95	.70	NA
Confidence in quick recovery from a disaster (N = 980)	2.10	.71	NA
***Social Cohesion***			
Close-knit neighborhood (N = 968)	2.10	.74	.71
Willing to help neighbors (N-977)	1.81	.61	.75
Generally don’t get along with each other (N = 977)	3.09	.61	.59
Do not share same values (N = 933)	2.79	.73	.57
Can be trusted (N = 960)	1.99	.66	.73
***Informal Social Control***			
Do something if children skipping school (N = 958)	2.09	1.03	.64
Do something if spray-painting graffiti (N = 974)	1.60	.89	.74
Scold child showing disrespect to adult (N = 937)	2.38	1.01	.57
Break up fight (N = 975)	1.74	.92	.67
Organize to keep open fire station (N = 959)	1.61	.86	.59
***Social Exchange***			
Favors for each other (N = 972)	2.02	.96	.78
Watch over neighbor’s property (N = 983)	1.80	.96	.63
Ask each other for advice (N = 959)	2.55	1.03	.65
Have parties (N = 985)	2.43	1.02	.66
Visit each other’s homes (N = 991)	2.02	.92	.76

#### Control variables

The control variables included in this analysis were storm impact, neighborhood SES, and a set of demographic variables (i.e., age, race, gender, income, and education). Extant research suggests that both a traumatic community-level event [7,32] and community-level SES [6,33] can have an effect on community-level properties such as resilience.

*Storm impact on neighborhood*. Respondents were asked: “Thinking about your current neighborhood, how seriously was your neighborhood affected by Superstorm Sandy? Would you say your neighborhood was extremely affected, very affected, moderately affected, only a little affected, or not at all affected? [Half of respondents received the response options in reverse order]. Answers were coded from 1 (not at all affected) to 5 (extremely affected).

*Neighborhood SES*. The 12 neighborhoods in our study were split into two SES groups based on median annual household income, $73,600 (six above and six below).

*Age*. All respondents were asked: “In what year were you born?”

*Race*. All respondents were asked a series of questions about race and ethnicity. First, respondents were asked: “Are you Hispanic, Latino, or Spanish origin?” Those who said yes were coded as Hispanic, and then asked: “In addition to being Hispanic, Latino, or Spanish origin what race or races do you consider yourself to be?” Respondents who said they were not Hispanic, were asked: “What race or races do you consider yourself to be?”

*Household Income*. All respondents were asked a series of questions about their household income. First, all respondents were asked “Does your total household income fall below $50,000, or is it $50,000 or higher?” Based on their answers, respondents were then asked which income group included their total household income when given four different options.

*Gender*. Phone and field interviewers coded respondents’ gender, but if uncertain interviewers asked: “Are you male or female?”

*Education*. All respondents were asked: “What is the last grade of school you completed?”

### Analysis

First, we used ordered logistic regression to assess the direct effects of collective efficacy (social cohesion and informal social control) and social exchange on both perceptions of disaster preparedness and beliefs about recovering from a major disaster in the future. For each of the six models, we conducted a Brant test to assess the proportional odds assumption for ordinal logistic regression. The overall chi square p-value was greater than .10 for all the models except preparedness with social cohesion, in which the p-value = .02. Based on this result, we ran a generalized ordered logistic model and the results were consistent with those of the ordinal logistic model. Regression models controlled for household income, race, age, education, gender, neighborhood SES and the impact of Superstorm Sandy on the neighborhood. Second, we examined whether these effects differed based on the SES of a neighborhood. We conducted a median split of neighborhoods based on income, and ran separate ordered logistic regression models for people in high and low SES neighborhoods. We also illustrate these results graphically. Finally, we examined whether storm impact moderates the association between social resource and resilience (i.e., perceptions of preparedness and recovery). We included an interaction term between storm impact and each social resource for people in high and low SES neighborhoods. All analyses used the weighted data and adjusted for the complex sample design of the survey in estimating variance.

## Results

Social cohesion, informal social control and social exchange were all associated with higher levels of neighborhood disaster preparedness (see Table 2). People who live in communities with higher social cohesion (coefficient = .73, p <.001), informal social control (coefficient = .53, p <.001), and social exchange (coefficient = .69, p <.001) were more likely to believe their neighborhoods were extremely or very well prepared for a disaster, when controlling for other covariates in the model. For example, those people living in communities with high social cohesion were approximately twice as likely as those in communities with lower social cohesion to believe their neighborhoods were well prepared for a disaster (odds ratio = 1.99, p <.001). Storm impact was significant only in the model that incorporated social exchange; results suggest that the worse the storm’s impact, the less prepared residents feel after the storm.

**Table 2:** Social cohesion, social control and social exchange and their associations with beliefs about disaster preparedness.

Disaster Preparedness	Model 1	Model 2	Model 3
Social Cohesion	0.73*** (0.12)		
Informal Social Control		0.53*** (0.11)	
Social Exchange			0.69*** (0.09)
Household Income	0.03 (0.05)	0.06 (0.05)	0.08 (0.05)
Hispanic	-0.25 (0.36)	-0.55 (0.37)	-0.47 (0.31)
Black	0.30 (0.49)	-0.08 (0.43)	-0.19 (0.41)
Other Race	-0.37 (0.44)	-0.33 (0.46)	-0.21 (0.46)
Age	-0.08 (0.09)	-0.02 (0.09)	0.05 (0.09)
Education	-0.01 (0.06)	0.02 (0.06)	-0.05 (0.06)
Gender	-0.09 (0.21)	-0.16 (0.21)	-0.16 (0.21)
Storm Impact	-0.18 (0.10)	-0.17 (0.09)	-0.26** (0.09)
Neighborhood SES	0.23 (0.22)	0.22 (0.23)	0.34 (0.22)
N	686	680	720
F	5.11	4.15	7.81

* p<0.05,

** p<0.01,

*** p<0.001.

Social cohesion, informal social control and social exchange were all also associated with an increase in confidence that people’s neighborhood will recover quickly from a major disaster in the future (see Table 3). People who live in communities with higher social cohesion (coefficient = .35, p <.01), informal social control (coefficient = .27, p <.05), and social exchange (coefficient = .42, p <.001) were at greater odds of being extremely or very confident their neighborhoods will recover quickly from a major disaster in the future, controlling for demographic factors and storm impact. For example, those living in communities with high levels of social exchange were about 1.5 times more likely to be confident their neighborhoods will recover quickly from a major disaster than those in communities with lower levels of social exchange (odds ratio = 1.53, p <.001). In contrast to the results for preparedness, storm impact was significant in all three models; results indicate that beliefs about recovery were shaped by the level of Sandy’s effect on the community.

**Table 3:** Social cohesion, social control and social exchange and their associations with beliefs about recovery from future disasters.

Disaster Recovery	Model 1	Model 2	Model 3
Social Cohesion	0.35** (0.13)		
Informal Social Control		0.27* (0.11)	
Social Exchange			0.42*** (0.11)
Household Income	0.05 (0.05)	0.03 (0.05)	0.04 (0.05)
Hispanic	-0.21 (0.34)	-0.53 (0.35)	-0.38 (0.31)
Black	0.18 (0.40)	0.04 (0.40)	-0.04 (0.41)
Other Race	-0.21 (0.35)	-0.04 (0.30)	-0.07 (0.34)
Age	-0.06 (0.08)	-0.08 (0.09)	-0.04 (0.08)
Education	-0.01 (0.07)	0.00 (0.07)	-0.02 (0.07)
Gender	0.25 (0.21)	0.16 (0.21)	0.21 (0.21)
Storm Impact	-0.38*** (0.09)	-0.36*** (0.09)	-0.43*** (0.09)
Neighborhood SES	0.19 (0.25)	0.15 (0.26)	0.09 (0.22)
N	696	685	727
F	3.06	3.10	3.99

* p<0.05,

** p<0.01,

*** p<0.001.

### Estimates by Neighborhood Socioeconomic Status (SES)

Social cohesion, informal social control, and social exchange were all associated with perceptions of greater disaster preparedness across neighborhoods (see Table 4). Social cohesion was associated with greater disaster preparedness for residents of both low SES (coefficient = .70, p <.001) and high SES neighborhoods (coefficient = .74, p <.001). Informal social control was also associated with greater disaster preparedness for residents of both low SES (coefficient = .49, p <.001) and high SES neighborhoods (coefficient = .57, p <.01). Likewise, social exchange was associated with greater disaster preparedness for residents of both low SES (coefficient = .82, p <.001) and high SES neighborhoods (coefficient = .49, p <.001).

**Table 4:** Social resources and their associations with beliefs about disaster preparedness for low and high SES neighborhoods.

Disaster Preparedness	Social Cohesion Models		Informal Social Control Models		Social Exchange Models	
	Low SES	High SES	Low SES	High SES	Low SES	High SES
Social Cohesion	0.70*** (0.17)	0.74*** (0.17)				
Informal Social Control			0.49*** (0.13)	0.57** (0.21)		
Social Exchange					0.82*** (0.13)	0.49*** (0.14)
Household Income	0.05 (0.06)	-0.02 (0.07)	0.10 (0.07)	-0.01 (0.08)	0.13 (0.06)	-0.02 (0.07)
Hispanic	-0.27 (0.49)	-0.35 (0.56)	-0.24 (0.46)	-1.18 (0.60)	-0.44 (0.45)	-0.61 (0.48)
Black	0.38 (0.47)	-1.10 (1.12)	0.07 (0.46)	-0.77 (1.05)	-0.02 (0.45)	-1.76 (1.00)
Other Race	-0.19 (0.52)	-0.65 (0.64)	-0.00 (0.58)	-0.84 (0.55)	0.12 (0.54)	-0.53 (0.62)
Age	-0.15 (0.13)	0.04 (0.12)	-0.07 (0.13)	0.07 (0.14)	-0.02 (0.12)	0.16 (0.12)
Education	-0.10 (0.07)	0.11 (0.10)	-0.05 (0.07)	0.13 (0.11)	-0.15 (0.07)	0.05 (0.10)
Gender	-0.24 (0.30)	0.06 (0.31)	-0.28 (0.30)	0.01 (0.31)	-0.44 (0.31)	0.10 (0.30)
Storm Impact	-0.16 (0.13)	-0.19 (0.14)	-0.19 (0.13)	-0.13 (0.14)	-0.28* (0.13)	-0.19 (0.13)
N	320	366	330	350	354	366
F	3.51	3.70	3.36	2.99	6.55	2.54

p<0.05,

** p<0.01,

*** p<0.001.

Social cohesion was associated with increased confidence in recovery among residents of low SES neighborhoods (coefficient = .34, p <.05), but not high SES neighborhoods (coefficient = .32, ns; see Table 5). Informal social control was associated with greater confidence in a quick recovery among residents of low SES neighborhoods (coefficient = .29, p <.05), but not high SES neighborhoods (coefficient = .22, ns). Likewise, social exchange was associated with greater confidence in a quick recovery among residents of low SES neighborhoods (coefficient = .54, p <.001), but not high SES neighborhoods (coefficient = .19, ns).

**Table 5:** Social resources and their associations with beliefs about recovery from future disasters for low and high SES neighborhoods.

Disaster Recovery	Social Cohesion Models		Informal Social Control Models		Social Exchange Models	
	Low SES	High SES	Low SES	High SES	Low SES	High SES
Social Cohesion	0.34* (0.16)	0.32 (0.18)				
Informal Social Control			0.28* (0.12)	0.22 (0.22)		
Social Exchange					0.59*** (0.13)	0.16 (0.19)
Household Income	0.06 (0.07)	0.06 (0.06)	0.04 (0.07)	0.06 (0.07)	0.06 (0.07)	0.04 (0.06)
Hispanic	0.25 (0.46)	-0.78 (0.50)	-0.06 (0.44)	-1.18* (0.58)	-0.07 (0.43)	-0.70 (0.54)
Black	0.64 (0.45)	-3.54* (1.40)	0.45 (0.45)	-3.44** (1.54)	0.34 (0.47)	-3.80** (2.38)
Other Race	0.22 (0.44)	-1.01 (0.56)	0.46 (0.26)	-1.08 (0.58)	0.36 (0.42)	-0.95 (0.61)
Age	-0.04 (0.12)	-0.05 (0.11)	-0.05 (0.12)	-0.09 (0.12)	-0.02 (0.12)	-0.03 (0.11)
Education	0.02 (0.08)	-0.04 (0.10)	0.03 (0.08)	-0.04 (0.11)	0.00 (0.08)	-0.03 (0.10)
Gender	0.25 (0.28)	0.16 (0.31)	0.26 (0.29)	0.00 (0.32)	0.19 (0.28)	0.13 (0.30)
Storm Impact	-0.50*** (0.13)	-0.25 (0.13)	-0.45*** (0.12)	-0.25 (0.13)	-0.57*** (0.13)	-0.25* (0.13)
N	326	370	332	353	357	370
F	3.22	2.98	3.44	2.61	5.38	2.17

p<0.05,

** p<0.01,

*** p<0.001.

**Fig 2:**
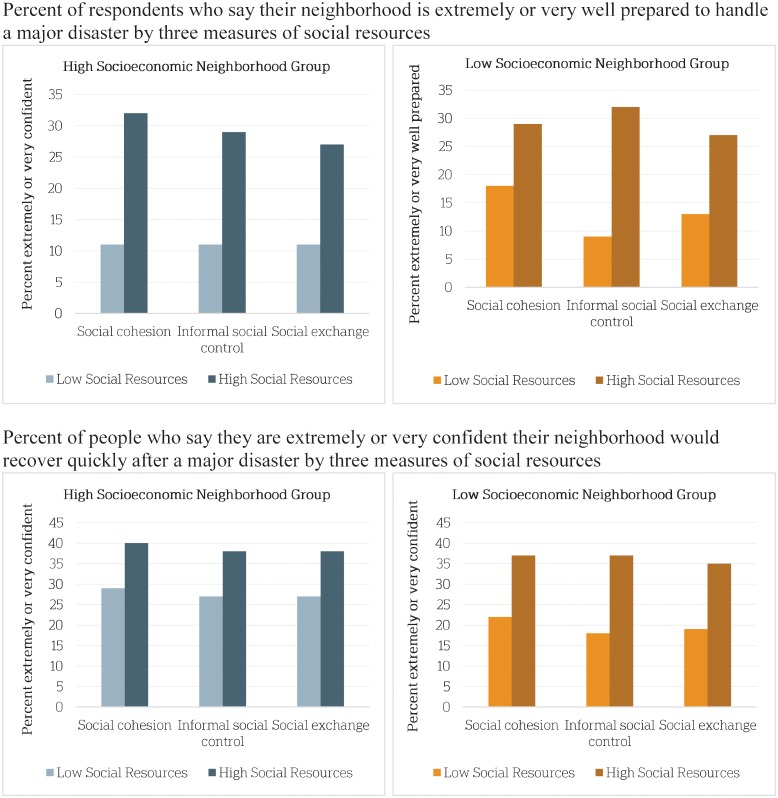
Social resources and resilience by neighborhood SES. The 12 neighborhoods in the survey are divided into high and low SES based on median household income (six neighborhoods each either above or below $73,601 a year). Social cohesion, informal social control and social exchange are all measured for each individual respondent with a factor score derived from their answers to a series of related questions. These factor scores are then split at the median (high and low, as above).

In general, results suggest that social resources have a stronger relationship with resilience (i.e., preparedness and recovery) in neighborhoods characterized as lower SES. Results in Fig 2 illustrate these relationships for both low and high SES neighborhoods. For each of the three measures of social resources, respondents were split at the median so those with factor scores above the median were classified in the high social resources group and those below the median were classified in the low social resources group. In all neighborhoods, those in the high social resources groups were more likely than those in the low social resources groups to believe their neighborhoods are prepared for a disaster and confident their neighborhoods would recover quickly. Results also suggest that high informal social control was associated with larger increases in confidence of recovery, on average, in low SES neighborhoods (105 percent increase) than in high SES neighborhoods (41 percent increase). Likewise, high social exchange was associated with larger increases in confidence of recovery, on average, in low SES neighborhoods (84 percent increase) than in high SES neighborhoods (41 percent increase). Consistent with earlier results, storm impact was more consequential to beliefs about recovery than preparedness and its influence was greater in low SES communities. We tested interaction effects between social resources and neighborhood SES and found no statistically significant moderating associations. Nevertheless, we do find evidence of association for low SES neighborhoods.

### Estimates of Storm Impact on Social Resources

Results suggest that storm impact may moderate the association between social resources and either preparedness or recovery in limited circumstances. In particular, the interaction models indicated that social resources tend to have more positive associations with preparedness and recovery when the storm impact to a neighborhood was less severe. The interaction term was significant for social exchange on perceptions of recovery (p< .05) in low SES neighborhoods and for social cohesion on perceptions of recovery (p< .05) in high SES neighborhoods (Tables 6 and 7).

**Table 6:** Social resources and their associations with beliefs about disaster preparedness for low and high SES neighborhoods.

Disaster Preparedness	Social Cohesion Models		Informal Social Control Models		Social Exchange Models	
	Low SES	High SES	Low SES	High SES	Low SES	High SES
Storm Impact*Social Resource	-.12 (.13)	-.12 (.14)	-.02 (.11)	.04 (.14)	-.19 (.12)	-.03 (.11)
Social Cohesion	1.16* (.49)	1.25* (.60)				
Informal Social Control			.57 (.38)	.41 (.55)		
Social Exchange					1.51*** (.42)	.62 (.42)
Household Income	.04 (.06)	-.03 (.07)	.10 (.07)	.01 (.08)	.12 (.06)	-.02 (.07)
Hispanic	-.25 (.49)	-.28 (.56)	-.23 (.46)	-1.17 (.60)	-.42 (.44)	-.61 (.49)
Black	.34 (.47)	-1.16 (1.17)	-.07 (.46)	-.72 (1.05)	-.01 (.44)	-1.78 (1.01)
Other Race	-.20 (.52)	-.54 (.64)	-.00 (.58)	-.86 (.55)	.18 (.54)	-.52 (.60)
Age	-.15 (.13)	.04 (.12)	-.07 (.13)	.07 (.14)	-.01 (.12)	.16 (.12)
Education	-.10 (.08)	.01 (.10)	-.05 (.07)	.12 (.11)	-.09 (.07)	.04 (.10)
Gender	-.25 (.30)	.07 (.31)	-.28 (.30)	.01 (.31)	-.45 (.29)	.10 (.30)
Storm Impact	-.20 (.14)	-.19 (.14)	-.19 (.13)	-.14 (.14)	-.31 (.14)	-.19 (.13)
N	320	366	330	350	354	366
F	3.35	3.51	3.05	2.75	6.41	2.49

p<0.05,

** p<0.01,

*** p<0.001.

**Table 7:** Social resources and their associations with beliefs about recovery from future disasters for low and high SES neighborhoods.

Disaster Recovery	Social Cohesion Models		Informal Social Control Models		Social Exchange Models	
	Low SES	High SES	Low SES	High SES	Low SES	High SES
Storm Impact*Social Resource	-.12 (.14)	-.36* (.14)	-.15 (.10)	-.19 (.14)	-.23* (.12)	-.04 (.14)
Social Cohesion	.78 (.46)	1.92** (.61)				
Informal Social Control			.81* (.37)	.99* (.50)		
Social Exchange					1.44** (.42)	.31 (.50)
Household Income	.05 (.07)	.05 (.06)	.03 (.06)	.06 (.07)	.04 (.07)	.05 (.06)
Hispanic	.27 (.46)	-.59 (.50)	-.01 (.44)	-1.19 (.60)	-.05 (.43)	-.70 (.54)
Black	.61 (.45)	-3.71 (1.43)	.49 (.45)	-3.77 (1.70)	.35 (.46)	-3.84 (1.41)
Other Race	.20 (.45)	-.69 (.61)	.46 (.36)	-1.00 (.54)	.43 (.44)	-.93 (.59)
Age	-.04 (.12)	-.05 (.11)	-.04 (.13)	-.010 (.12)	-.01 (.12)	-.03 (.11)
Education	.02 (.09)	-.02 (.10)	.03 (.08)	-.03 (.10)	-.02 (.08)	-.03 (.10)
Gender	.25 (.29)	.21 (.30)	.26 (.29)	-.01 (.32)	.19 (.28)	.13 (.30)
Storm Impact	-.54 (.13)	-.27 (.13)	-.49 (.13)	-.22 (.13)	-.63 (.13)	-.24 (.14)
N	326	370	332	353	357	370
F	3.25	3.54	3.33	2.51	5.16	1.99

* p<0.05,

** p<0.01,

*** p<0.001.

## Discussion

Examining the region two years after Superstorm Sandy made landfall, we learn that residents feel better equipped to withstand another natural disaster, both in terms of preparedness and confidence in recovery, if they believe their communities exhibit the social connectedness to respond to it. This connectedness, reflected in measures of social cohesion, informal social control, and social exchange, predicts both perceptions of preparedness and confidence in recovery. Whether in a high or low SES neighborhood, all three measures relate to perceptions of preparedness. In contrast, confidence in recovery varies across economic status of the neighborhood; these social resources are more predictive of recovery for those in low SES neighborhoods. This suggests both that preparedness and confidence in recovery capture different aspects of the disaster experience, and that low SES neighborhoods may more readily rely on these social resources in the context of recovery (so imagine they are a necessary condition of rebuilding). Not surprisingly, storm impact is also influential although, not in keeping with expectations, social resources tend to have more positive associations with preparedness and recovery when the storm impact to a neighborhood was less severe.

Why do the results related to social resources and SES vary for confidence in recovery but not in preparedness? Perhaps preparedness is viewed as more contingent on residents coming together toward collective goals. Recovery, by comparison, may be seen as subject to a mix of social, economic, and political resources (so no matter how effective a community may be, a full recovery requires a combination of actors and resources that might lie outside the purview of neighborhood residents) [20,23]. Community-level wealth may provide a certain sense of confidence that mobilization could occur without sufficient social connections in place, and that ties to external and/or formal sources (e.g., government entities) could be galvanized effectively. Variation by neighborhood SES is an important component of our results. Our finding that social resources, in general, are more consequential in low SES communities is consistent with previous research that suggests that collective efficacy and social exchange may deliver a set of resources in their own right and may, in certain circumstances, provide as much or perhaps more than economic resources [4].

Our results suggest that examining collectively efficacy (social cohesion and informal social control) and social exchange separately was fruitful; these social processes did have differential associations with preparedness and recovery. Further, examining the two components of collective efficacy revealed that, in the general case, social cohesion tended to be more impactful than informal social control. Social cohesion and social exchange are most similar to one another and relatively consistent in their association with preparedness and recovery. This may be due to the nature of these two concepts, that they are more oriented toward help-seeking (and help-granting) behavior.

Our moderation analysis suggests that storm impact shapes expectations about recovery rather than preparedness (although results are modest). It may be that, beyond a certain point, the severity of the storm requires infrastructure support of a nature that may not readily draw on the social resources described in this paper. Recovery may be harder to imagine than effective preparation, particularly when one’s frame of reference draws from a more challenging storm-related context.

What can we do to enhance communities so that preparedness and recovery are viewed as possible? One investment may be for municipalities to allocate resources to establish or bolster infrastructure that facilitates social interaction. This suggests urban design akin to that described by Jane Jacobs [34] and some contemporary urban planners [35–37]; mixed use urban space that encourages residents to pass one another as they engage in routine activities facilitates connectedness and trust, potentially providing a reservoir of assistance in time of need. It also suggests programmatic responses such as block parties and other activities aimed at collective engagement that, again, provide the opportunity for social interaction. Creating opportunity for social engagement in typical and non-disaster time periods suggests that residents will form bonds free of the pressure that arises from emergency conditions. Facilitating social connections, and harnessing the capital that arises from them, may create resilience at the community-level that is comparable to, or exceeds, an investment in physical infrastructure.

Our findings should be interpreted in light of our study limitations. First, we only focus on twelve neighborhoods in detail, not the entire area of New York and New Jersey affected by Superstorm Sandy. That said, we did impose some variation by design, ensuring we would be able to concentrate on areas that varied by economic well-being and recovery status. Focusing our resources in this manner allowed for an in-depth treatment of recovery and resilience. Second, it might have been helpful to ask about resilience directly. Resilience is a useful but broad construct; in this instance we elected to focus on markers of resilience—preparedness and recovery—that speak to resilience, and may be precursors of it, but are perhaps more concrete and interpretable for respondents. Third, we acknowledge that a higher response rate might provide for more robust analyses. Still, the response rate is similar to or higher than those of most contemporary public affairs surveys. Fourth, other data that reflect preparedness and recovery during Superstorm Sandy are very limited but future work on climate-related disasters might benefit from external sources of information related to recovery and resource provision. Finally, selection into neighborhoods is always a concern in neighborhood-based research and we cannot account for all factors that inform residential choice or mobility. The vast majority of those surveyed (88 percent) reported living at the same address at the time of Superstorm Sandy while 4 percent were living in the same neighborhood, 5 percent were living somewhere else affected by the storm, and 2 percent were living somewhere not affected by the storm. These data provide some confidence that our respondents are able to effectively report on their current neighborhoods of residence, but selection factors that may have contributed to their initial residential choice cannot be fully accounted for in these analyses.

This work focuses on perceptions of preparedness and likelihood of recovery, two key features of resilience at the neighborhood-level. Perceptions shape action, and the extent to which residents perceive their neighborhoods as prepared or better able to recover likely leads to further investments in community and its social fabric. Low SES communities, in particular, appear to benefit from collective efficacy and social exchange at the neighborhood level, but communities across the economic spectrum feel more prepared when these resources are present. These findings suggest that programs and policies meant to ready communities for the next natural disaster must first consider an investment in the social infrastructure of their neighborhoods.
